# Voluntary interruption of psychotic pregnancy: Use of antipsychotic drugs in a bioethical case

**DOI:** 10.1192/j.eurpsy.2021.1610

**Published:** 2021-08-13

**Authors:** J. Galvañ, I. Angélico

**Affiliations:** 1 Psychiatry, Hospital Universitario de La Princesa, Madrid, Spain; 2 Psychiatry, Hospital Universitario Son Espases, Palma de Mallorca, Spain

**Keywords:** psychosis, bioethica, antipsychotic, pregnancy

## Abstract

**Introduction:**

Decisions about use and safety of antipsychotic drugs during pregnancy it’s been a controversial issue in psychiatric practice because of the difficult finding the good choice, ethically and medically.

**Objectives:**

To provide an example of a real case to shed light about the psychopharmacological and ethical management of the situation helping a psychotic patient to make a voluntary decision.

**Methods:**

Expose a clinical case of a patient in a psychiatric institution for several psychotic symptoms who we discover she’s pregnant during her hospitalization and treatment process. She is a 36 years old single woman who shows disorganized maniac psychotic behavior including disinhibition, promiscuity, persecutory and symbolic delusional ideas, self-surrender and insomnia. She’s admitted against her will in a University Hospital, being transferred to a Psychiatric Hospital with risperidone (2mg/24h) and clonazepam 2mg (2mg/24h). She had a positive pregnant test. Receiving the patient, we made an updated bibliographical review about use of antipsychotic during pregnancy, consult with the patient’s family and hospital legal advice’s service and coordinate with Gynecology’s service. The patient was ambivalent about the decision conditioned by her symptoms.

**Results:**

We decide to optimize drugs to olanzapine (until 30mg/24h) during the first week not using mood stabilizers because of malformations risk, with a great amelioration of symptoms, experiencing a back to reality with a coherent speech and eutimia, deciding a voluntary interruption of pregnancy.

**Conclusions:**

Psychosis in pregnancy can be a bioethical challenge wich must be management according to science (practice clinical guidelines point olanzapine as a choice to be considered) and woman’s will.

